# Rapid Response to Drive COVID-19 Research in a Learning Health Care System: Rationale and Design of the Houston Methodist COVID-19 Surveillance and Outcomes Registry (CURATOR)

**DOI:** 10.2196/26773

**Published:** 2021-02-23

**Authors:** Farhaan Vahidy, Stephen L Jones, Mauricio E Tano, Juan Carlos Nicolas, Osman A Khan, Jennifer R Meeks, Alan P Pan, Terri Menser, Farzan Sasangohar, George Naufal, Dirk Sostman, Khurram Nasir, Bita A Kash

**Affiliations:** 1 Houston Methodist Houston, TX United States

**Keywords:** COVID-19, SARS-CoV-2, data science, data curation, electronic health records, learning health system, databases, factual

## Abstract

**Background:**

The COVID-19 pandemic has exacerbated the challenges of meaningful health care digitization. The need for rapid yet validated decision-making requires robust data infrastructure. Organizations with a focus on learning health care (LHC) systems tend to adapt better to rapidly evolving data needs. Few studies have demonstrated a successful implementation of data digitization principles in an LHC context across health care systems during the COVID-19 pandemic.

**Objective:**

We share our experience and provide a framework for assembling and organizing multidisciplinary resources, structuring and regulating research needs, and developing a single source of truth (SSoT) for COVID-19 research by applying fundamental principles of health care digitization, in the context of LHC systems across a complex health care organization.

**Methods:**

Houston Methodist (HM) comprises eight tertiary care hospitals and an expansive primary care network across Greater Houston, Texas. During the early phase of the pandemic, institutional leadership envisioned the need to streamline COVID-19 research and established the retrospective research task force (RRTF). We describe an account of the structure, functioning, and productivity of the RRTF. We further elucidate the technical and structural details of a comprehensive data repository—the HM COVID-19 Surveillance and Outcomes Registry (CURATOR). We particularly highlight how CURATOR conforms to standard health care digitization principles in the LHC context.

**Results:**

The HM COVID-19 RRTF comprises expertise in epidemiology, health systems, clinical domains, data sciences, information technology, and research regulation. The RRTF initially convened in March 2020 to prioritize and streamline COVID-19 observational research; to date, it has reviewed over 60 protocols and made recommendations to the institutional review board (IRB). The RRTF also established the charter for CURATOR, which in itself was IRB-approved in April 2020. CURATOR is a relational structured query language database that is directly populated with data from electronic health records, via largely automated extract, transform, and load procedures. The CURATOR design enables longitudinal tracking of COVID-19 cases and controls before and after COVID-19 testing. CURATOR has been set up following the SSoT principle and is harmonized across other COVID-19 data sources. CURATOR eliminates data silos by leveraging unique and disparate big data sources for COVID-19 research and provides a platform to capitalize on institutional investment in cloud computing. It currently hosts deeply phenotyped sociodemographic, clinical, and outcomes data of approximately 200,000 individuals tested for COVID-19. It supports more than 30 IRB-approved protocols across several clinical domains and has generated numerous publications from its core and associated data sources.

**Conclusions:**

A data-driven decision-making strategy is paramount to the success of health care organizations. Investment in cross-disciplinary expertise, health care technology, and leadership commitment are key ingredients to foster an LHC system. Such systems can mitigate the effects of ongoing and future health care catastrophes by providing timely and validated decision support.

## Introduction

As of December 31, 2020, over 90 million COVID-19 cases had been confirmed worldwide [1]. The COVID-19 pandemic has tested the limits of human resilience, leading to innovation in several facets of clinical and academic medicine [2,3]. Prior to the pandemic, the health care industry had already been on the precipice of a digital revolution driven by big data, machine learning, and artificial intelligence for a long time. The pandemic brought to bear a dire need for investment in robust health data infrastructures and pipelines (DIPs) such that barriers and latency to gather, assimilate, validate, and share data widely and swiftly can be minimized or eliminated [4]. Establishing and maintaining robust clinical DIPs are resource intensive and require a cross-disciplinary approach. Effective utilization of health care data to drive clinical and operational decision-making, in the context of a true learning health care (LHC) system, warrants organizational commitment—both at the technical level and as a behavioral paradigm shift.

For several health care organizations, the urgency to synthesize epidemiological and clinical evidence for understanding the rapidly evolving COVID-19 pandemic has underscored the need for innovation in two sperate yet overlapping processes: (1) the review process for approval of COVID-19–related minimal risk research while maintaining stringent federal and institutional standards of human-subject research and (2) the critical and fundamental need to establish a reliable and valid DIP to serve as the backbone for swift and accurate reporting. Organizations with an LHC focus and infrastructural investment are highly likely to be agile and adaptive to such rapidly developing needs and thus be on the forefront of combating health care catastrophes.

This paper provides an overarching account of how the needs for data accessibility, rapid research, and reliable reporting evolved in the face of the COVID-19 pandemic across a large health care system and its associated research enterprise. Both the health care system and research enterprise are located in a very populous and diverse US metropolis (Houston, Texas) that became a hub of the second wave of the COVID-19 pandemic during the summer of 2020. We share our experiences of the methodology implemented for addressing the aforementioned needs, which included (1) assembling and leveraging expertise from interdisciplinary and multispecialty teams; (2) listing considerations that include regulation and ethics of COVID-19 research; (3) leveraging organizational aspects of coordinating and harmonizing cross-institutional data and research needs; and finally, (4) the development, technical design, and implementation of the Houston Methodist COVID-19 Surveillance and Outcomes Registry (CURATOR). All these items are in line with the health care system’s institutional goal of fostering a true LHC.

## Methods

### Implementation Setting

#### Greater Houston Metropolitan Area and the Houston Methodist System

Like other large metropolitan areas across the United States, the Greater Houston area experienced a rise in COVID-19 cases in early March 2020. The Greater Houston Metropolitan Statistical Area—officially designated by the Office of Budget and Management as “Houston–The Woodlands–Sugar Land”—is the fifth most populous area in the United States, with an approximate population of 7 million [5]. The Greater Houston area is also considered to be one of the nation’s most ethnically diverse regions [6]. Harris County, whose county seat is Houston, is the third largest county in the United States in terms of population, whereas the city of Houston is the fourth most populated US city [5]. On March 1, 2020, there was only 1 known and officially reported case of COVID-19 in the Harris County/Houston area, which increased to almost 6000 over an 8-week period. The first surge, which peaked in mid-April, saw later a 3- to 4-fold increase in cases by early July 2020 [7]. The total number of COVID-19 cases in the 9-county Houston Metropolitan Statistical Area is estimated to be over 315,000, as of December 31, 2020 [8].

Houston Methodist, along with its centers of excellence in cancer, heart and vascular, digestive disorders, neurology, orthopedics and sports medicine, and transplant, and an academic affiliation with Weill Cornell Medicine and New York Presbyterial Hospital (New York, USA), comprises one flagship tertiary care hospital (Houston Methodist Hospital) and six large community hospitals, with an additional long-term care hospital, spanning across the Greater Houston area. Additionally, the system has an expansive emergency medicine and ambulatory health care network including an Accountable Care Organization [9]. Houston Methodist Research Institute and Houston Methodist Academic Institute lead the basic science, translational, clinical and epidemiological outcomes research, and training portfolio for the system [9,10].

#### COVID-19 Clinical, Administrative, and Research Data Needs at Houston Methodist

Houston Methodist became the clinical hub for COVID-19 in the Greater Houston area and the first in the United States to perform plasma transfusion as part of COVID-19 treatment [11]. As soon as Houston Methodist started testing for COVID-19 and providing care to infected patients, the urgent need for validated, ongoing data on COVID-19 treatment and outcomes mandated institutional prioritization. Data requirements came from three broad categories of stakeholders. First, the frontline care teams needed data to support the clinical decision-making process; second, hospital administration and leadership needed data to efficiently manage hospitals’ resources and outwardly communicate to the public; and third, clinical researchers needed data to explore innumerable important research questions. Anecdotal information on potentially beneficial therapies and effective management algorithms started flowing in, and there was a dire need to “validate” treatment efficacies and management modalities in the local context. Administrators needed quick and reliable metrics on not only the number of COVID-19 cases but also precise projections on mortality rates, length of stay, days in intensive care units (ICUs), and utilization of critical hospital resources such as ventilators and personal protective equipment. In addition, several centers of excellence and clinical departments immediately needed access to data of patients with COVID-19 to analyze important disease patterns and consequences on their respective patient populations. Consequently, there was an overwhelming outpouring of proposals and research ideas that started flowing to the Institutional Review Board (IRB). To provide rapid responses while preserving research integrity, two system-wide subcommittees were established: the Clinical Trials Task Force, which was tasked to evaluate proposals for therapeutic clinical trials, and the Retrospective Research Task Force (RRTF), which was set in place to facilitate the review and coordination of all observational (retrospective and prospective) research across the system. Many authors of this manuscript (FSV, HDS, BAK, SLJ, KN, and JRM, along with representation from the IRB and corporate and research information technology departments) constituted the membership of the RRTF.

### Current Implementation of Electronic Health Record System

The past decade has seen a dramatically increased propagation of electronic health records (EHRs) in the United States. This phenomenon was largely promoted through large US government-initiated programs to encourage the adoption of EHRs in routine practice (eg, Meaningful Use, Certification Commission for Health Information Technology; inducements in the Affordable Care Act; Health Information Technology for Economic and Clinical Health Act in 2009; and the mandatory submission of quality measures electronically). Although certain benefits of EHRs are undeniable, they are most often designed and implemented with the administrative end-user in mind. In most cases, a system with a focus on administration, with streamlined billing and coding features, is not adapted for the assimilation of research data. Coincidentally, this same system contains a plethora of social, demographic, and medical information on thousands of patients in one location and is quite possibly one of the largest underutilized resources in modern medical research. However, at the time of the cusp of the COVID-19 pandemic, many health care facilities, including our own, lacked EHR add-ons that would allow for a rapid assimilation of research datasets. As the COVID-19 pandemic ensued, our research infrastructure faced an unprecedented need for validated datasets to support clinical trials and observational studies. Hence, to support research activities based on EHR, the RRTF decided to set up the Houston Methodist CURATOR. The goal of CURATOR is to serve as a unified, longitudinal, cross-institutional registry for COVID-19 data, to fulfill ongoing and long-term observational research data needs and enable availability of data for planning of prospective clinical trials.

## Results

### Structure, Workflow, and Output of the Houston Methodist COVID-19 RRTF

**Figure 1:**
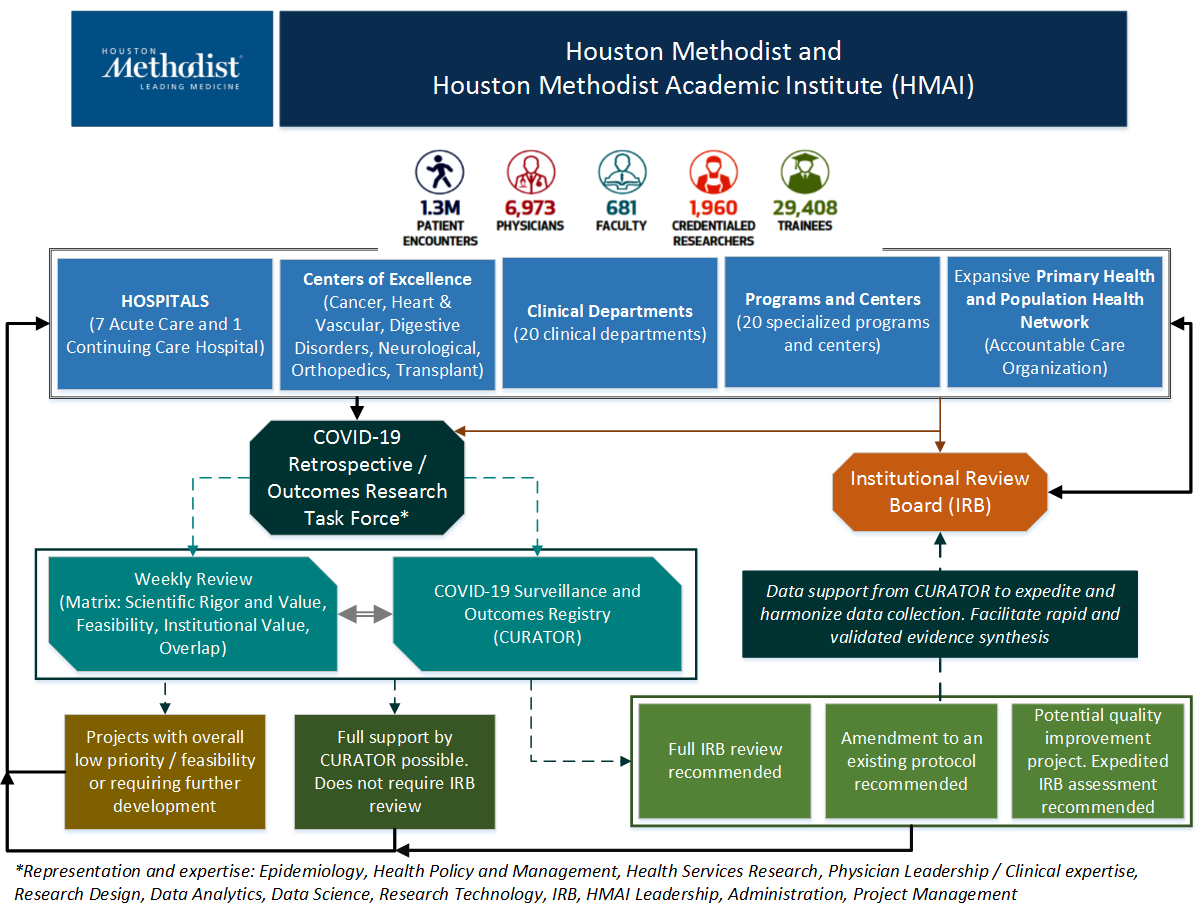
Schematic representation of the COVID-19 task force and the Houston Methodist COVID-19 Surveillance and Outcomes Registry (CURATOR) placement in the organizational context.

The RRTF was established on March 20, 2020 as a pre-IRB step after the institutional leadership effected a decision to accelerate the internal review, triage, and operationalization of a growing number of observational research protocols that were received by the IRB. The overarching clinical and academic structure of the Houston Methodist system, and the RRTF process framework in relation to the IRB, is schematically represented in Figure 1. The top two panels represent the organizational distribution of several physicians, physician scientists, translational and epidemiological scientists, and trainees spread across various hospitals, centers of excellence, clinical departments, programs and specialized centers, and an expansive primary care network. The solid black arrows represent communication pathways between investigators across this clinical and research enterprise and various elements of the COVID-19 RRTF and the Houston Methodist IRB. The RRTF initially reviewed all protocols related to COVID-19 and communicated back to the investigators directly in situations where the projects had opportunities for further development, were not technically sound, or did not require a full IRB review (see bottom-left brown and dark green text boxes in Figure 1). All other protocols, with specific comments and recommendations were forwarded to the IRB for a full evaluation (see bottom-right green text boxes in Figure 1). The Center for Outcomes Research (COR) at Houston Methodist Research Institute was tasked to set up the charter and workflow for the RRTF. The COR leadership team assembled the preliminary process documentation for the RRTF and a team comprising experts in epidemiology, health systems research, health policy, clinical domains, data sciences, information technology, and research regulation.

To accelerate and prioritize review of the influx of COVID-19–related protocols, all protocols received either directly or indirectly via the IRB are evaluated independently by RRTF members with a prioritization matrix and discussed on a weekly basis. The outcome of the RRTF process is communicated back to the investigators. During the extensive review activities developed by the multidisciplinary RRTF team, it was observed that many promising observational studies required similar data resources, leading to the development of a central COVID-19 data infrastructure to expedite research output for all scientists engaged in COVID-19 research [12]. For this purpose, the RRTF decided to develop and actively maintain a registry for COVID-19 surveillance and intrahospital outcomes as a key tangible output of its research-acceleration function. The design, data aspects, and front-end of this registry are addressed in the next section.

### The Houston Methodist CURATOR Protocol

#### CURATOR Design and Cohorts

The Houston Methodist CURATOR protocol was developed by the COR leadership and was approved by the Houston Methodist IRB on April 11, 2020. The CURATOR database comprises two cohorts. The first cohort includes all individuals who were tested (regardless of the test result) for COVID-19 at any Houston Methodist location (hospital or a free-standing clinical establishment) with any of the SARS-CoV-2 diagnostic tests, including antigen tests and the polymerase chain reaction test, or for SARS-CoV-2 serology. All clinical encounters dating back to June 2016 are included without a prospective end date. This means that the database includes, for each patient, the records of all prior (pretesting) and subsequent (posttesting) clinical encounters (ie, hospitalizations, emergency department or primary care visits, laboratory tests, imaging reports, medications, and specialists care) that happen either as standard of care, or as a part of systematic long-term follow-up (such as follow-up in specialized COVID-19 recovery clinics). The second cohort comprises COVID-19 patients who were managed at Houston Methodist facilities but originally tested elsewhere. Like the first cohort, any instances of clinical encounters prior to hospitalization or postacute care are included. More recently, CURATOR’s protocol has been amended to include all individuals who have received or will be receiving a COVID-19 vaccine, regardless of their COVID-19 status.

**Figure 2:**
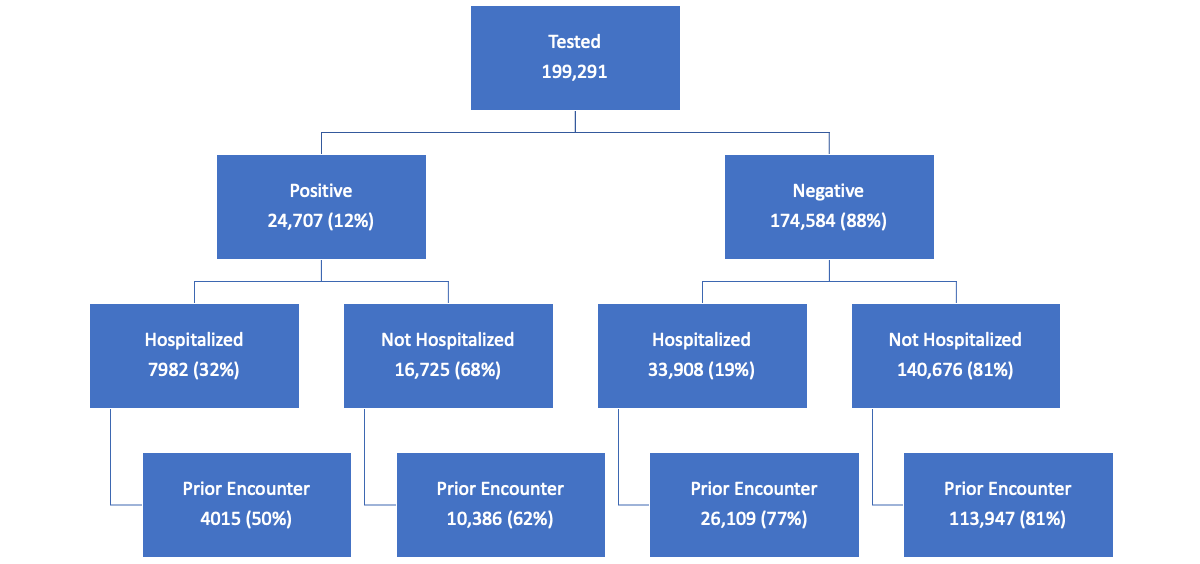
Schematic representation of the total number and proportion of individuals who underwent COVID-19 testing, those who tested negative and positive, and those who were hospitalized with prior clinical encounters, based on data from COVID-19 Surveillance and Outcomes Registry (CURATOR).

The design elements of CURATOR allow for two salient aspects that strengthen methodological approaches in hypothesis generation and testing. First, by tracking records per patient, CURATOR creates a longitudinal array of individuals’ health status. Availability of data from clinical encounters prior to testing and/or hospitalization permits granular, time-dependent, and accurate risk stratification for comorbidities based on longitudinally obtained medications, imaging, and laboratory test results, rather than cross-sectional documentation of comorbid and pre-existing conditions at the time of COVID-19–related hospitalization or clinical encounter. Similarly, the information obtained from subsequent (post–COVID-19) encounters will provide information on recovery and outcomes. The second unique design element of CURATOR is the readily available data from a control population. By including data on all tested individuals, capturing retrospective and prospective clinical encounters of individuals who tested positive as well as those who tested negative for COVID-19, a large number of potential controls are available in CURATOR for hypothesis testing. For instance, matched case-control studies or prospective cohorts for incidence-based analyses can be used when developing COVID-19–related hypotheses. Figure 2 provides an updated schematic of the total number and proportion of individuals who underwent COVID-19 testing, those who tested positive or negative, and those who were hospitalized with at least one prior clinical encounter in the CURATOR.

#### CURATOR Data Elements and Data Structure

CURATOR is a relational structured query language database that is directly populated with the back-end data originating in one of the market-leading EHR vendors in the United States. The COR was uniquely equipped to undertake the creation and implementation of CURATOR with data scientists experienced in working with EHRs. The initial effort was to assemble back-end data by grouping disparate but related information from different locations within the EHR. Then, the database was iteratively refined to create meaningful tables and views of the data in an analytic data set that can be useful for researchers. The EHR back-end data refreshes every 24 hours based on live instances of the institutional EHRs.

The extract, transform, and load (ETL) procedures from EHR to the CURATOR database have been designed, developed, and implemented by a multispecialty team of COR scientists (big data and data science leads, epidemiologists, and physicians), data scientists, data engineers, and application analysts. In parallel, other ETL processes across the institution have also been simultaneously implemented by business intelligence teams to support clinical, operational, and administrative decision-making. The CURATOR team has undertaken an ongoing validation process with the business intelligence counterparts to streamline the ETL process and assess internal validity of COVID-19–related data across the system. Targeted smaller COR–business intelligence teams have been working together to this end. Updates and issues resulting from this cooperation are reviewed in weekly, biweekly, and monthly meetings. Currently, CURATOR is being populated with 1004 data items aggregated and organized across 87 tables and views. CURATOR is updated weekly by using an automated ETL process designed and implemented by the COR Big Data & Artificial Intelligence team. This process has been optimized to achieve efficiency and version control.

#### Current Data

Currently, CURATOR contains extracted information for approximately 200,000 individuals, of whom approximately 25,000 tested positive for COVID-19, with approximately 14 million hospital encounters. For each patient, basic demographic (eg, age, sex, zip code, geocoding, marital status, and education level), ethnic identity (ethnicity and race), and baseline health (eg, BMI, IDC-10 code Charlson comorbidities, clinical morbidities, and immunizations) data are included. In addition, the CURATOR database includes the time-detailed ordering and results of laboratory tests, imaging, and procedures for each patient. For instance, the laboratory tests include cultures, real-time reverse transcription polymerase chain reaction tests, and SARS-CoV-2 antigen and antibody tests, among 3709 other COVID-19–related and unrelated tests for a total of approximately 76 million laboratory tests and results. The imaging results include multi-region computed tomography (CT)–coupled angiograms, abdomen, chest and heart CT scans, echocardiograms, and multi-vessel interventional radiology, among 1977 other distinct imaging results, with approximately 1.8 million results in the database. The procedures include isolation; intraosseous infusions; red blood cell transfusion through peripheral veins; introduction of sera, toxoids, and vaccines into muscles; insertion of a tunnel vascular devices into the patient’s chest; prone status; and 10,063 other procedures, with a total of approximately 400,000 procedures in the database. Furthermore, CURATOR contains time-resolved registries of the medications ordered (for inpatients and outpatients) and administered (inpatients), results on clinical trials, and the outcomes of each patient, including details on discharge status, discharge location, length of stay, oxygen therapy, ICU stays, the usage of mechanical ventilators, extracorporeal membrane oxygenation and endotracheal intubation, among many other variables informed by numerous research proposals that have traversed the RRTF process. The CURATOR database is continually growing to address wider research needs.

#### CURATOR Integration with Other Internal and External Data Sources

CURATOR’s design and implementation allows for seamless integration with other unique and siloed sources of big data across Houston Methodist. The virtual ICU (vICU) provides continuous, digitalized intensivist coverage for over 300 ICU beds at Houston Methodist. This remote ICU monitoring environment with embedded advanced telehealth capabilities captures real-time continuous physiological data on all ICU patients (including those with COVID-19) and provides an opportunity to develop predictive analytical tools to proactively identify critical risk factors and anticipate patient decompensation. The vICU platform at Houston Methodist was rapidly expanded following the COVID-19 pandemic [13]. Thus, vICU information is being integrated into CURATOR to broaden research perspectives and enrich the case histories with streaming physiologic data captured in real-time.

Additionally, Houston Methodist hosts one of the very few advanced translational imaging centers in the United States. This image center includes one of the most powerful 7-Tesla magnetic resonance imaging machines available. These advanced imaging modalities are rapidly being leveraged for the assessment, prognostication, and prediction of the effect of COVID-19 on pulmonary, cardiac, and neurological tissues. The outcomes of advanced COVID-19 imaging analyses will also be integrated into CURATOR.

Furthermore, Houston Methodist utilizes an innovative digital care navigation and data collection system for patient communication, education and awareness, and capturing patient-reported outcomes measures in postacute and long-term care setting (CareSense, MedTrak, Inc [14]). By using automated yet customized phone calls, text messages, emails, and app notifications, patients on various digital pathways are followed up with overarching goals to provide effective transition of care, promote safe recovery, and prevent complications. Success of these pathways has been previously reported and similar pathways are actively used for patients with COVID-19 [15]. The structure of CURATOR allows for seamless integration of the data sources obtained from the digital care navigation system.

Finally, CURATOR data is linkable via direct or probabilistic matching with external sources such as state-wide or national claims and administrative data sources. Certain derived data elements, such as area deprivation index [16], are now integrated into the routine workflow of CURATOR data updates.

#### Regulation and Governance (Annual Audit, Review, and Stewardship)

The CURATOR protocol is governed and regulated by the Houston Methodist IRB. The protocol, training, and delegation logs, data governance policies, and data release and sharing procedures have been approved by the IRB, are maintained and updated by COR project management, and are subject to annual IRB audits. The governance committee comprises the COR leadership. All projects proposing to utilize CURATOR are subject to an independent review by the IRB. Projects led by Houston Methodist investigators that do not warrant sharing of protected health information may be exempt from an IRB review. However, these assessments are undertaken by the RRTF that has IRB representation. To date, CURATOR actively supports more than 30 IRB-approved COVID-19 research protocols across Houston Methodist (Textbox 1).

**Current list of projects at Houston Methodist approved by the Institutional Review Board and supported by COVID-19 Surveillance and Outcomes Registry (CURATOR).**

**COVID-19 projects categorized by clinical discipline**

- **Cardiology**
  - Echocardiographic Findings in COVID-19 Patients
  - Cardiovascular Magnetic Resonance Imaging of Myocardial Damage in COVID-19 Patients
  - Vascular Disease and Complications of COVID-19
  - Troponin Elevation and Myocardial Infarction in COVID-19 Patients
  - Statin Therapy, Lipid Control, and Severe Illness in COVID-19 Among Patients With Cardiovascular Disease
  - Area Deprivation Index and Indicators of Severe COVID-19 Among Patients With Cardiovascular Disease

- **Neurology**
  - Stroke Outcomes Among COVID-19 Patients
  - Cognitive Outcomes Among COVID-19 Patients

- **Infectious disease**
  - Epidemiology of COVID-19
  - Biospecimens Related to COVID-19

- **Public health or disparities**
  - Race and Ethnic Disparities in SARS-CoV-2 Susceptibility
  - Race and Ethnic Disparities in COVID-19 Hospitalization and Mortality
  - Sex Differences in COVID-19 Outcomes
  - Characteristics and Outcomes of COVID-19 Across Various Pandemic Phases
  - Medication Outcomes Surveillance for COVID-19
  - ICU Ethics for COVID-19

- **Surgery**
  - Emergency Surgical Volumes during COVID-19 Pandemic
  - Outcomes among Transplant and Non-transplant Recipients with COVID-19
  - Surgery during COVID-19 Pandemic

- **Critical care**
  - Corticosteroid Use in COVID
  - Hydroxy Chloroquine Use and Outcomes in COVID-19
  - Proning Associated Outcomes in COVID-19
  - Tocilizumab Use and Outcomes in COVID-19
  - NISQIP and COVID-19
  - COVID-19 Treatment Algorithms and Outcomes

- **Rehab or physical therapy**
  - Physical Therapy in COVID-19 ICU

#### Front-end User Interface

Data availability and democratization is a key component of the acceleration function of CURATOR. End-users can rapidly test hypotheses and identify feasible research lines based on preliminary studies using the database. Nonetheless, the access of end-users to data must be IRB-regulated, and processes and procedures to protect the data set from mishandling must be implemented. For this purpose, the CURATOR registry contains a web-accessible front-end that allows the end-user access to IRB-approved parts of the database via customizable, interactive charts. The charts are developed on static copies of CURATOR that are updated weekly; hence, accidental information disarrangement or system resources overconsumption is practically avoided. Our end-goal is to make the front-end of CURATOR available as a research tool across the health sciences communities. The front-end will also provide a seamless web-based communication platform between investigator teams, CURATOR management, and the IRB.

## Discussion

### Principal Findings

In this paper we describe how demanding data requirements were addressed by an administratively situated, EHR-integrated data structure for rapidly updated surveillance and outcomes data in the context of the COVID-19 pandemic.

The initial protocol was approved in less than 4 weeks from submission to the IRB review due to the critical need, via intensely responsive investigator–IRB communication. The scope involved several components that trigger particular IRB deliberation, including data pulled from various sources retrospectively and prospectively, with identifiers intact, under waiver of consent and Health Insurance Portability and Accountability Act (HIPAA) authorization, with future data banking planned. To simplify that deliberation and expedite the launch of a functional registry, components such as data sharing and data linking with potential external partners, sub-study personnel and scope of sub-studies, and secondary use of research data (including follow-up contact with patients in the registry) were relegated to future amendments or addenda to be reviewed by the IRB at a later date. This afforded the researchers’ envisioned data governance committee time to convene and establish thoughtful policies on these matters; most importantly, the transparent promise of future amendments presents the IRB with information in an amount and at the time when necessary for implementation.

In our experience, it takes a unique multidisciplinary team, empowered by close contact with executive leadership, and a balance between ethics/rigor and speed during a pandemic to be able to drive impactful and meaningful observational research. At Houston Methodist, the integration of this team as a pre-IRB approval task force allowed us to design tools for fast-tracking research proposal triage, review, and operationalization. Furthermore, close contact with the hospital’s leadership was key for the rapid dissemination of the RRTF duties, competences, and activities. In addition, the creation of a centralized task force unit allowed the RRTF to identify a set of common data elements across research proposals by performing transversal analysis, thus allowing the data teams to begin data extraction in parallel with the IRB process to expedite the availability of the data to various research teams once they have secured IRB approval for their study. The insight into what was “in the research pipeline” allowed our data team to deliver data into the hands of researchers much more quickly than if IRB approval and data gatekeeping had been a serial process, as it ordinarily is.

Having one unified database as a single source of truth (SSoT) allowed us to focus resources on developing a database maintained with the highest standards. Additionally, the multidisciplinary nature of the team allows us to continuously enlarge the CURATOR database by looking at the SARS-CoV-2 pandemic from different angles and for different applications. Finally, the transition of CURATOR from a registry to a live source for hypothesis testing and research-line identification is being carried out by developing a front-end for this database. The availability of this front-end will not only reduce the querying loads to the back-end maintainers of the CURATOR database, but it will also help practitioners and researchers shorten the hypothesis test-validation cycle, leading to improved practice and research performance, respectively.

In addition to creating an SSoT, we aligned our approach and efforts with several other established principles of driving an effective digitization of health care industry [17]. First, CURATOR aims to break down data silos and create true functional interoperability between heterogenous data sources such as the traditional EHR, vICU, CareSense, and imaging data warehouses across the system. Second, we continue to evaluate and develop the analytical maturity of our informatics pipelines. As an example, the CURATOR infrastructure provides a concrete context and platform to utilize leading cloud-based technologies for analysis of continuous waveform data, develop machine learning and artificial intelligence models for image synthesis, and harness Natural Language Processing for some of the applications described below as current limitations. Third, by generating a validated cross-linkage between CURATOR and other business intelligence–driven data process across Houston Methodist, we aligned CURATOR’s goals with that of the organization at large. Even though CURATOR has been set up as a research-oriented data resource, harmonization across the institution adds value and helps in engaging a wider array of stakeholders and resource allocation for continued support. Fourth, collaborating with the IRB and institutional leadership, we have set up robust governance structures that are clearly communicated and disseminated. Finally, our front-end interface provides data insight, data exploration, and communication tools that essentially facilitate regulated yet efficient data democratization and is a platform for developing further stake-holder driven applications.

The CURATOR model has significant implications for future research. In addition to providing a COVID-19–specific research platform, the CURATOR model also establishes a replicable DIP framework across several other clinical disciplines, particularly in the context of an LHC system. We believe that the focus of our institutional leadership on fostering a true LHC system enabled us to successfully resurrect and implement this infrastructure during a global pandemic. A systematic effort to set up a similar framework across cardiology and neurology service domains is underway and significant investments have been made across other clinical domains. The CURATOR model, although catalyzed by the COVID-19 pandemic, is not a “one-and-done” project; instead, it is an ardent representation of a data centric health care organization that has poised itself to lead medicine and health care delivery and overcome health care digitization challenges of the future.

### Comparisons to Prior Work

In the wake of the COVID-19 pandemic, the need for validated data sources has been appreciated widely across the health care industry. Broadly, two approaches have been adopted and reported in literature. First, existing registries and data warehouses have been modified to include data elements pertaining to COVID-19. In most cases, such resources are clinical domain–driven, such as the American Heart Association’s Get With The Guidelines Registries [18], the American Academy of Orthopedic Surgeon’s Registry [19], the American College of Surgeons COVID-19 Registry [20], the American College of Radiology COVID-19 Imaging Research Registry [21], and the American Academy of Dermatology Association COVID-19 Registry [22]. This approach capitalizes on an existing network of participating organizations and has an advantage of a fairly well-established data pipeline and governance structure. However, this approach is specific to individual clinical domains and is therefore of limited utility to a wider array of stakeholders.

The second approach entails establishing dedicated data repositories for COVID-19 research, agnostic to other clinical domains. Data and information regarding such registries are, however, limited. Theoretically, these could be single or multi-institution endeavors. The Innovative Support for Patients With SARS-CoV-2 Infections Registry (INSPIRE) has been registered with ClinicalTrials.gov with a primary outcome of ascertaining incidence of myalgic encephalomyelitis or chronic fatigue syndrome across 8 institutions [23]. The INSPIRE investigators propose to enroll 3600 patients with COVID-19 and 1200 controls over a 2-year period. Other emerging examples of single-center COVID-19 registries include the Stanford University COVID-19 registry [24], Rice University COVID-19 Registry [25], and Johns Hopkins COVID-19 Precision Medicine Analytics Platform Registry (JH-CROWN) [26]. Dedicated COVID-19 registries have the strength of providing a platform for comprehensive analyses of COVID-19–related hypotheses, regardless of patients with pre-existing comorbidities or other clinical conditions. However, considerable de novo ETL efforts may be required to set up such resources. Furthermore, as the evidence indicates, establishing cross-institutional partnerships will take considerable additional effort in creating common data definitions models, harmonizing data processes, and setting up regulatory and governance structures.

CURATOR exemplifies a single, large health care institutional COVID-19 registry. However, given the pre-existing institutional commitment and investment in an LHC system, it was established at a rapid pace and, as we have discussed, conforms to several of the fundamental principles of health care digitization. Without much information published on other institutional COVID-19 registries, a direct head-to-head comparison is not feasible. However, CURATOR capitalizes on several unique data sources and currently supports COVID-19 projects across several domains along with work already published from CURATOR data and its associated resources across Houston Methodist [7,27-32].

### Limitations

Although the architecture of CURATOR was designed and automated to retrieve new and updated data in a near-real-time implementation, it is limited to a single-center, longitudinal medical history record. In its current iteration, CURATOR cannot capture clinical encounter information from systems outside of Houston Methodist. In the context of a global pandemic, this is a significant limitation. However, the CURATOR leadership currently partners with local, regional, national, and international consortia, which provides an ongoing opportunity to establish common data element models for harmonization with external data sources. There are also significant challenges with other incomplete, yet highly relevant data (eg, presenting symptoms data is largely unstructured and incomplete with regards to salient elements such as timing, progression and severity of symptoms, as well as palliative measures). Future implementation of natural language processing pipelines is envisioned as a solution. CURATOR, like all retrospective registries, relies on “samples of convenience,” and hence suffers from a certain degree of selection and information bias. Systematic selection of COVID-19 patients and planned follow-up in COVID-19 recovery clinics across Houston Methodist will minimize the influence of such potential bias. Finally, quantifiable assessment of true impact of CURATOR on reducing research timelines across our organization is not currently possible due to the limitation of resources that would be needed to perform a comparison across historical data or collect specific metrics on investigators’ perspectives. However, CURATOR metrics are being actively monitored and documented and such assessments would be possible in future. Despite these limitations, CURATOR and similar efforts are powerful tools in finding the signal in the noise when confronted from every angle with the unknown, as we are, during the outbreak of any novel pathogen.

### Conclusions

In the face of rapidly evolving COVID-19 pandemic, the health care industry’s challenge of meaningful digitization has been exacerbated. Developing a data-driven, clinical, operational, and research decision-making strategy is paramount to the success of health care organizations. We share our experience of how a large, tertiary care health care organization and its research enterprise rapidly adapted to this challenge and created COVID-19–centric mechanisms of efficient and validated decision-making across a complex health care enterprise. The cross-disciplinary expertise, investment in health care technology, and leadership commitment are key ingredients to establish and foster an LHC system. Such systems, if optimally developed, can mitigate the effects of ongoing and future health care catastrophes by providing timely and validated decision support mechanisms.

## Abbreviations

COR: Center for Outcomes Research
CT: computerized tomography
CURATOR: COVID-19 Surveillance and Outcomes Registry
DIP: data infrastructure and pipeline
EHR: electronic health record
ETL: extract, transform, and load
ICU: intensive care unit
INSPIRE: Innovative Support for Patients With SARS-CoV-2 Infections Registry
LHC: learning health care system
RRTF: retrospective research task force
SSoT: single source of truth
vICU: virtual intensive care unit
